# Acceptance of smoking cessation support and quitting behaviours of women attending Aboriginal Maternal and Infant Health Services for antenatal care

**DOI:** 10.1186/s12884-021-03569-z

**Published:** 2021-01-26

**Authors:** Justine B. Daly, Sarah Dowe, Belinda Tully, Flora Tzelepis, Christophe Lecathelinais, Karen Gillham

**Affiliations:** 1 Hunter New England Population Health, Hunter New England Local Health District, Locked Mail Bag 10, Wallsend, New South Wales 2287 Australia; 2 Hunter Medical Research Institute, Locked bag 1000, New Lambton, New South Wales 2305 Australia; 3 NSW Rural Doctors Network, 53 Cleary Street, Hamilton, New South Wales, Australia; 4 School of Medicine and Public Health, University of Newcastle, University Drive, Callaghan, New South Wales 2308 Australia

**Keywords:** Smoking cessation, Pregnancy, Antenatal care, Aboriginal

## Abstract

**Background:**

Acceptance of smoking cessation support during antenatal care and associated quitting behaviours of pregnant Aboriginal women or women having an Aboriginal baby has not been investigated. This study aimed to determine, among pregnant women who smoke and attended AMIHS for their antenatal care:

1. The acceptance of smoking cessation support, factors associated with acceptance and barriers to acceptance;
2. The prevalence of quitting behaviours and factors associated with quitting behaviours.

**Methods:**

A cross-sectional telephone survey of women who attended 11 AMIHSs for their antenatal care during a 12 month period in the Hunter New England Local Health District of New South Wales.

**Results:**

One hundred women contacted consented to complete the survey (76%). Of those offered cessation support, 68% accepted NRT, 56% accepted follow-up support and 35% accepted a Quitline referral. Participants accepting NRT had greater odds of quitting smoking at least twice during the antenatal period [OR = 6.90 (CI: 1.59–29.7)] and those reporting using NRT for greater than eight weeks had six times the odds of quitting smoking for one day or more [OR = 6.07 (CI: 1.14–32.4)].

**Conclusions:**

Aboriginal women or women having an Aboriginal baby who smoke make multiple attempts to quit during pregnancy and most women accept smoking cessation support when offered by their antenatal care providers. Acceptance of care and quitting success may be improved with increased focus on culturally appropriate care and enhanced training of antenatal care providers to increase skills in treating nicotine addiction and supporting women to use NRT as recommended by treatment guidelines.

**Supplementary Information:**

The online version contains supplementary material available at 10.1186/s12884-021-03569-z.

## Background

Smoking during pregnancy is associated with multiple obstetric and fetal complications including placenta previa, placental abruption, low birth weight, preterm birth, still birth and miscarriage [1]. Pregnant women who quit smoking can however significantly reduce their risk of such negative pregnancy outcomes, with cessation earlier in pregnancy resulting in the best outcomes for both mother and baby [1–3].

In Australia, as in most high income countries, smoking rates during pregnancy have steadily declined [4]. These overall reductions have not been reflected in smoking rates of pregnant Aboriginal women, which remain disproportionately high compared to non-Aboriginal women (45% vs 12%) [5]. The impact of colonisation, racism, socio-economic disadvantage, marginalisation and the resulting stressful life circumstances are all likely contributors to this disparity [6, 7].

Systematic review evidence and best practice clinical guidelines for smoking cessation in the antenatal period recommend that health care professionals provide behavioural counselling, recommend Nicotine Replacement Therapy (NRT) (when behavioural counselling alone is insufficient), and provide follow-up cessation support, including referrals for telephone counselling, to facilitate smoking cessation during pregnancy [8–10]. In New South Wales, Australia (NSW), Aboriginal Maternal and Infant Health Services (AMIHS) provide culturally appropriate antenatal care to Aboriginal women and women having an Aboriginal baby (who do not identify as Aboriginal themselves), from conception to 8 weeks postpartum [11]. Such services present a unique opportunity to provide smoking cessation support to pregnant Aboriginal women and women having an Aboriginal baby as a part of routine antenatal care [12]. Limited evidence is available that examines whether smokers in this population are amenable to and will engage with all elements of best practice smoking cessation support during their antenatal care. Research to date has primarily focussed on describing Aboriginal women’s experiences of smoking cessation care during pregnancy [13] and their views on the perceived helpfulness of various support strategies [14, 15]. No studies have examined actual acceptance of all elements of best practice smoking cessation support by pregnant Aboriginal women or women having an Aboriginal baby in antenatal settings, or explored the factors associated with acceptance of such support. Furthermore, no data exist that describe which smoking cessation support strategies may be associated with quitting behaviours amongst this group of women. This information is important to understand whether current best practice approaches are acceptable to and may facilitate smoking cessation within this priority population group [15, 16].

This study aimed to determine, among pregnant women who smoke and attended AMIHS for their antenatal care:

1. The acceptance of smoking cessation support, factors associated with acceptance and barriers to acceptance;
2. The prevalence of quitting behaviours and factors associated with quitting behaviours.

## Methods

### Study design and setting

A cross-sectional survey was undertaken between September and November 2015, of women who attended 11 AMIHSs for their antenatal care during a 12 month period in the Hunter New England Local Health District (HNELHD) of NSW. The HNELHD includes a major metropolitan centre, as well as regional and remote communities, with 4% of the population identifying as Aboriginal [17]. The AMIHS service delivery model includes home visiting and community based clinics and care is provided by a team consisting of a midwife and an Aboriginal Health Worker [11]. Aboriginal Health Workers are pivotal in supporting the provision of culturally safe and appropriate care and facilitating engagement of women with health services. This role includes advocacy, support, liaison, disease prevention and health promotion. At the time of the survey HNELHD AMIHSs were involved in the delivery of Quit for New Life, a NSW Ministry of Health funded initiative aiming to increase best practice smoking cessation care delivery including the provision of free NRT [18].

### Sample and recruitment

Women were identified from ObstetriX, an antenatal medical records database used by public antenatal services in NSW. Women were eligible to participate if they attended an AMIHS antenatal ‘booking-in’ appointment between January and December 2014 and self-reported that they were a smoker during this ‘booking in’ visit. Women whose record indicated a negative pregnancy outcome such as miscarriage or stillbirth were excluded from the sample.

### Procedure

An Aboriginal Advisory group oversaw the development of the telephone survey. This group provided advice on culturally appropriate language and cultural sensitivity of survey content and processes. The survey was reviewed by a focus group of AMIHS Aboriginal staff and clients before being pilot tested with 6 AMIHS clients and Aboriginal staff from the Population Health Unit.

Eligible women were mailed a letter inviting them to participate in a Computer Assisted Telephone Interview (CATI) regarding the smoking cessation support and information they received during their antenatal care. Women were subsequently contacted by an interviewer to obtain verbal informed consent to complete a 20 minute telephone survey. Only female interviewers were utilised, there was the option of an Aboriginal interviewer if preferred and all non-Aboriginal interviewers completed cultural awareness and respect training.

Ethics approval was provided by the Aboriginal Health & Medical Research Council of NSW Ethics Committee (1075/15) and the Hunter New England Human Research Ethics Committee (14/12/10/5.11), including an Aboriginal Health Impact Statement through the HNELHD Aboriginal Health Unit.

### Measures

#### Offer and acceptance of smoking cessation support and barriers to acceptance

Women were asked if the AMIHS team had assessed their smoking status, offered them a Quitline referral, offered NRT and offered follow-up support (‘yes’, ‘no’, ‘don’t know’) during their antenatal care. Women who indicated that they were offered these support strategies were asked if they accepted the offer (‘yes, ‘no’, ‘don’t know’). For those accepting NRT, information was collected on type (oral, patch) and duration of use. Those reporting declining the offer of support were asked their reasons for not accepting (‘Not interested in quitting’, ‘Did not think it would help me quit’, ‘Tried before and it did not work’, ‘Worried about safety’, ‘Feeling uncomfortable”, ‘Want to quit without assistance’).

#### Prevalence of quitting behaviours

Women were asked if during the time that they were seeing the AMIHS: whether they ever quit smoking for one day or more (‘yes’, ‘no’, ‘don’t know’), how many times they quit for 1 day or more and of those quit attempts made, what was the longest time they went without a cigarette.

#### Demographic and other characteristics

Information was collected regarding number of antenatal visits to AMIHS, current smoking status (‘daily’, ‘once a week’, ‘< once per week’, ‘not at all’), number of cigarettes smoked per day, number of other smokers in the household and whether there were rules regarding smoking in the home. Age, educational attainment, relationship status and Aboriginal origin were also collected. (Supplementary File 1).

### Analysis

Statistical analysis was completed using SAS software version 9.3. Outcomes of interest included acceptance of cessation support (Quitline; NRT; follow-up support), duration of NRT use (≤ 8 weeks, > 8 weeks), and quitting behaviours (Quitting for one day or more; number of times quit for one day or more; longest time without a cigarette). Chi-Square and Fisher’s Exact tests were initially performed to investigate the associations between those outcomes and participant characteristics, as well as the association between the quitting behaviour outcomes and each cessation support strategy. Variables significant at *p* < 0.25 were then included into a backward stepwise logistic regression model for each outcome of interest [19]. Non-significant variables were removed until all remaining variables were significant at *p* < 0.05.

## Results

### Sample

335 eligible women were identified from the ObstetriX database. Of these, 76 were disconnected numbers, 130 were unreachable and 29 did not wish to participate. A total of 100 women completed the survey resulting in a consent rate of 76% and response rate of 30%. Comparison of socio-demographic characteristics extracted from the ObstetriX database found there were no significant differences between women who participated in the survey (*n* = 100) and those who did not (*n* = 232) in terms of age and Socio-Economic Indexes for Areas (SEIFA) score. Women who did not participate in the study were more likely to live in an outer regional/remote area (*p* = 0.02) than women who completed the survey.

### Women’s characteristics

Seventy six percent of women identified as Aboriginal and the mean age was 26 years. At least half (53%) lived with a partner and 87% reported that they were current smokers. Sixty two percent of women lived with one or more smokers. Sixty nine per cent of women had attended five or more antenatal visits with the AMIHS. (Table 1).

**Table 1:** Participant characteristics (*N* = 100)

	n	%
*Age*		
< 25	43	43
25–34	46	46
35+	11	11
*Age of participants (years)*		
Mean (SD)	26.3 (5.48)	
Median (range Min-Max)	25.5 (18–39)	
*Aboriginal (N = 99)* ^a^		
Yes	76	76
No	23	23
*Highest Education Level Completed*		
Year 10 or less	62	62
Year 12/Diploma/University Degree	38	38
*Relationship Status (N = 97)*		
Single	46	47
Live with partner or married	51	53
*Number of antenatal visits to AMIHS (N = 98)*		
< 5 visits	30	31
5 or more visits	68	69
*Current Smoking Status*^b^		
Smoker	87	87
Non-Smoker	13	13
*Cigarettes per day (N = 85)*^b^		
1–5	18	21
6–10	41	48
11+	26	31
*Number of other smokers in the household*		
None	38	38
One or more	62	62
*Rules regarding smoking in the home*		
Complete Smoking bans	93	93
Partial Smoking Bans	7	7

^a^ The term Aboriginal is used to describe both Aboriginal and Torres Strait Islander Peoples. Within NSW Health, the term Aboriginal is generally used in preference to Aboriginal and Torres Strait Islander, in recognition that Aboriginal people are the original inhabitants of NSW. Missing data n = 1, declined to answer question

^b^At time of survey

### Offer and acceptance of smoking cessation support

Almost all women (98%) reported having their smoking status assessed by the AMIHS, with 86% reporting being offered Quitline, 69% being offered free NRT and 59% being offered follow-up support. Of those offered this care, 35% reported accepting a Quitline referral, 68% reported accepting NRT and 56% accepted follow-up support. Of those reporting accepting NRT, less than half (44%) reported using it for more than 8 weeks (Table 2).

**Table 2:** Proportion of AMIHS clients reporting offer and acceptance of smoking cessation support during their antenatal care (*N* = 100)

	All Clients N = 100		Aboriginal Clients ***N*** = 76		Non Aboriginal Clients N = 23	
	n	%	n	%	n	%
Smoking status assessed	98	98	74	97	23	100
Quitline referral offered	86	86	64	84	22	96
Quitline referral accepted *(N = 86)*	30	35	19	30	11	50
Follow-up cessation support offered	59	59	44	58	14	61
Follow-up support accepted (*N* = 59)	33	56	23	52	9	64
NRT Offered	69	69	49	64	19	83
NRT Accepted *(N = 69)*	47	68	32	65	14	74
Type of NRT Used *(N = 47)*^a^						
Oral	40	85	26	81	14	100
Patch	21	45	16	50	5	36
Duration of NRT Use *(N = 43)*^b^						
≤ 8 weeks	24	56	14	48	10	71
> 8 weeks	19	44	15	52	4	29

^a^Participants may have used more than one type of NRT

^b^ Missing data n = 4

### Factors associated with acceptance of smoking cessation support

Non-Aboriginal women (women having an Aboriginal baby who do not identify as Aboriginal themselves) had almost 3 times the odds of accepting a Quitline referral [OR = 2.75 (CI: 1.04–7.25)] and NRT [OR = 2.80 (CI: 1.01–7.75] compared to those who identified as Aboriginal. All women attending five or more antenatal visits at AMIHS had 3 times the odds of accepting NRT compared to those who attended less than five visits [OR = 3.03 (CI: 1.16–7.94)]. No factors were found to be associated with accepting follow up support (Table 3).

**Table 3:** Factors associated with acceptance of smoking cessation support

	**Quitline referral accepted** **n (%)**	**OR**	**95% CI**	**P**
Identify as Aboriginal				
Yes (*N* = 64)	19 (30%)	Referent		0.041
No (*N* = 22)	11 (50%)	2.75	1.04–7.25	
	**NRT Accepted** **n (%)**	**OR**	**95% CI**	**P**
Identify as Aboriginal				
Yes (*N* = 49)	32 (65%)	Referent		0.047
No (*N* = 19)	14 (74%)	2.80	1.01–7.75	
Number of antenatal visits to AMIHS				
< 5 visits (*N* = 15)	9 (60%)	Referent		0.024
≥ 5 visits (*N* = 53)	38 (72%)	3.03	1.16–7.94	

### Barriers to acceptance of smoking cessation support

The three most common reasons women reported for declining the offer of smoking cessation support was a lack of interest in quitting smoking (47%), belief that the support would not be helpful (22%) and wanting to quit without assistance (16%).

### Prevalence of quitting behaviours

Sixty three percent of women reported they had quit for one day or more during the antenatal period, with 66% quitting two or more times. Of those who quit for one day or more, the mean time without a cigarette was 3 months (*Median: 0.27: SD 5.01 Min 0.03 – Max 18),* and 35% reported abstaining from smoking for one month or more (Table 4).

**Table 4:** Self-reported quitting behaviours of AMIHS clients during antenatal care period

	All Clients N = 100		Aboriginal Clients N = 76		Non Aboriginal Clients N = 23	
	n	%	n	%	n	%
*Quit for 1 day or more during antenatal care*						
Yes	63	63	47	62	15	65
No	37	37	29	38	8	35
*Number of times quit for 1 day or more (N = 62)*						
Once	21	34	17	36	4	27
2 or more times	41	66	30	64	11	73
*Longest time without a cigarette (N = 63)*						
Under 1 month	41	65	30	64	10	67
1 month or more	22	35	17	36	5	33

### Factors associated with quitting behaviours

Women who reported using NRT for greater than eight weeks had six times the odds of quitting smoking for one day or more during the antenatal period compared to those who used NRT for eight weeks or less [OR = 6.07 (CI: 1.14–32.4)]. Women accepting NRT had greater odds of quitting smoking at least twice during the antenatal period compared to those who did not accept NRT [OR = 6.90 (CI: 1.59–29.7)]. No factors were found to be associated with longest time without a cigarette (Table 5).

**Table 5:** Factors associated with quitting behaviours

	**Quit for one day or more** **n (%)**	**OR**	**95% CI**	**P**
Duration of NRT use				
≤ 8 weeks (*N* = 24)	14 (58%)	Referent		0.035
> 8 weeks (N = 19)	17 (89%)	6.07	1.14–32.4	
	**Quit at least twice for 1 day or more** **n (%)**	**OR**	**95% CI**	**P**
NRT Accepted				
Yes (*N* = 32)	24 (75%)	6.90	1.59–29.7	0.035
No (*N* = 11)	4 (36%)	Referent		

## Discussion

To our knowledge this is the first study to use quantitative data to examine the acceptance of smoking cessation support and factors associated with quitting behaviours amongst pregnant Aboriginal women and women having an Aboriginal baby. The findings demonstrate high rates of acceptance of cessation support by this group of pregnant women when it is offered as a part of routine antenatal care. We found that the majority of women had made quit attempts during their pregnancy and many had attempted to quit multiple times. However few were able to sustain their quit attempts. Quitting behaviours were significantly associated with acceptance of NRT and using NRT for greater than 8 weeks.

Despite Aboriginal women being significantly less likely to accept NRT or a Quitline referral when compared to non-Aboriginal women, level of acceptance was very encouraging with 65% accepting NRT, 52% accepting follow-up cessation support and just over a third accepting a Quitline referral. This supports previous research reporting high levels of acceptability amongst pregnant Aboriginal women who smoke for the provision of free NRT and cessation support by midwives and Aboriginal Health Workers [13, 15], however lower acceptability of Quitline [15]. Even though Quitline was least favoured, the proportion of referrals accepted (30%) were at least 5 times higher than that previously reported for self-referrals in the general population [20]. Such findings indicate a high level of willingness amongst pregnant Aboriginal women who smoke to accept support to make changes to their smoking and suggest that recommended best practice strategies to support smoking cessation, specifically, behavioural support by health providers, free NRT and Quitline referrals, are acceptable to pregnant Aboriginal women when offered as a part of routine antenatal care.

The findings of lower rates of acceptance of smoking cessation support by Aboriginal women compared to non-Aboriginal women having an Aboriginal baby, suggests that there may be additional barriers to accepting smoking cessation support for Aboriginal women attending an AMIHS. Participants in the current study cited three main barriers to accepting support to quit smoking; lack of interest in quitting smoking, belief that the support would not be helpful and wanting to quit without assistance. These findings are consistent with a recent qualitative study where Aboriginal women reported an ambivalence to the need for support to quit, preferring to take an independent approach to quitting [13]. This indicates that further strategies are needed to successfully engage Aboriginal women in cessation support, such as behavioural counselling and NRT, to increase their chances of successfully quitting [8–10], and that the development of such strategies should be informed by Aboriginal women. Additional strategies to increase engagement of Aboriginal women with cessation support could include strengthening the role of Aboriginal Health Workers in the delivery of cessation support to enhance cultural safety and tailoring to local contexts [12], providing support to partners and family members who smoke [6, 21], and comprehensive training for antenatal care providers to increase confidence and skills in treating nicotine addiction [12, 22]. This aligns with and places particular emphasis on services providing culturally appropriate care.

Acceptance of NRT was found to be associated with attending 5 or more antenatal clinic visits, indicating that repeated contact with the service was associated with increased likelihood of acceptance of NRT. Such findings support best practice clinical guidelines for pregnancy that recommend antenatal care providers re-address smoking at every contact with smoking clients [8–10] and reinforces the unique opportunity provided by the antenatal visit schedule for provision of smoking cessation support.

The majority of women in this study who continued to smoke, reported making multiple attempts to quit, however just over one third of women successfully sustained a quit attempt for greater than one month. Those who accepted NRT were significantly more likely to report quitting multiple times, while those who used NRT for greater than 8 weeks, the recommended treatment course [23], were significantly more likely to have quit for one day or more. Research to date has failed to confirm the efficacy of NRT in supporting smoking cessation during pregnancy [24]. This lack of evidence of effect has been attributed to poor compliance with both dose and duration of treatment as well as insufficient dosage to adequately account for higher rates of nicotine metabolism during pregnancy [24, 25]. Our findings indicate that women using NRT were making multiple quit attempts but many were unable to achieve sustained abstinence. To improve the likelihood of sustaining quit attempts, more emphasis may need to be placed on improving adherence to NRT treatment guidelines as well as ensuring that women are receiving an adequate dose of nicotine to manage withdrawal and provide craving relief [26]. This could be facilitated via the development of clinical guidelines to support health care provider decision making and improved staff training regarding NRT. Further, in light of these findings and recent Cochrane review evidence that providing free NRT increases the proportion of smokers who attempt to quit, use smoking cessation treatment and succeed in quitting [27], services providing antenatal care to Aboriginal women and women having an Aboriginal baby should consider making free NRT available to support women with quitting.

The findings of this study should be considered in light of potential limitations. First, whilst 76% of women contacted consented to complete the survey, the overall response rate was 30%. Whilst this increases risk of selection bias, comparison of socio-demographic characteristics between participants and non-participants found no differences other than non-participants being more likely to live in outer regional/remote areas. Further, annual attendance data from internal clinic records for HNELHD AMIHSs, indicates that the proportion of Aboriginal clients completing the survey (76%) is very similar to the actual client profile [28]. Future studies focussing on pregnant Aboriginal women should consider utilising alternative approaches for survey delivery to improve response rates and participation by women located in rural and remote areas [29]. Second, the use of a self-report measure for quitting behaviours may have resulted in some misreporting. Future studies could consider the inclusion of biochemical validation to address this. Finally, the sample was restricted to one local health district and to women attending an AMIHS for their antenatal care, as such the generalisability of the results may be limited.

## Conclusions

The findings of this study suggest that a high proportion of pregnant Aboriginal women who smoke and attend AMIHS for their antenatal care, will accept smoking cessation support when offered and that recommended best practice strategies to support smoking cessation are acceptable to pregnant Aboriginal women. Factors which may increase acceptance of support include readdressing smoking and the offer of support at every contact with women as well as focussing on improving cultural appropriateness through involving Aboriginal women in the development of strategies to engage Aboriginal women in cessation support. Finally, there is a need to strengthen the role of Aboriginal Health Workers, as well as more comprehensive training of antenatal care providers to increase confidence and skills in treating nicotine addiction and supporting women to use NRT as recommended by treatment guidelines.

## Supplementary Information

**Additional file 1.**

## Data Availability

The dataset used during the current study is available from the corresponding author on reasonable request.

## Abbreviations

AMIHS: Aboriginal Maternal and Infant Health Service
HNELHD: Hunter New England Local Health District
NRT: Nicotine Replacement Therapy
NSW: New South Wales
CATI: Computer Assisted Telephone Interview
SEIFA: Socio-Economic Indexes for Areas

## References

[1] US Department of Health and Human Services (2014). The health consequences of smoking —50 years of progress: a report of the surgeon general.

[2] Liu B, Xu G, Sun Y (2020). Maternal cigarette smoking before and during pregnancy and the risk of preterm birth: a dose–response analysis of 25 million mother–infant pairs. PLoS Med.

[3] Kondracki AJ, Hofferth SL (2019). A gestational vulnerability window for smoking exposure and the increased risk of preterm birth: how timing and intensity of maternal smoking matter. Reprod Health.

[4] Australian Institute of Health and Welfare (2017). National Drug Strategy Household Survey 2016: detailed findings.

[5] Australian Institute of Health and Welfare (2016). Tobacco indicators: measuring midpoint progress—reporting under the National Tobacco Strategy 2012–2018.

[6] Gilligan C, Sanson-Fisher RW, D'Este C, Eades S, Wenitong M (2009). Knowledge and attitudes regarding smoking during pregnancy among Aboriginal and Torres Strait islander women. Med J Aust.

[7] Passey ME, Gale JT, Sanson-Fisher RW (2011). "It's almost expected": rural Australian Aboriginal women's reflections on smoking initiation and maintenance: a qualitative study. BMC Women Health.

[8] Chamberlain C, O’Mara-Eves A, Porter J, Coleman T, Perlen SM, Thomas J, McKenzie JE. Psychosocial interventions for supporting women to stop smoking in pregnancy. Cochrane Database Syst Rev. 2017;(2). Art. No.: CD001055. 10.1002/14651858.CD001055.pub5. 10.1002/14651858.CD001055.pub5 PMC6472671 28196405

[9] Zwar NRR, Borland R, Peters M, Litt J, Bell J, Caldwell B, Ferretter I (2011). Supporting smoking cessation: a guide for health professionals. Melbourne: The Royal Australian College of General Practitioners.

[10] New South Wales Ministry of Health Centre for Population Health (2014). Guidelines for the Management of Substance Use During Pregnancy Birth and the Postnatal Period. North Sydney, Australia.

[11] New South Wales Health (2006). NSW Aboriginal maternal and infant Health strategy evaluation: final report 2005*.* Sydney, Australia.

[12] Passey ME, Bryant J, Hall AE, Sanson-Fisher RW (2013). How will we close the gap in smoking rates for pregnant indigenous women?. Med J Aust.

[13] Bovill M, Gruppetta M, Cadet-James Y, Clarke M, Bonevski B, Gould GS (2018). Wula (voices) of Aboriginal women on barriers to accepting smoking cessation support during pregnancy: findings from a qualitative study. Women Birth.

[14] Bovill M, Bar-Zeev Y, Bonevski B (2020). Aboriginal Wingadhan Birrang (woman’s journey) of smoking cessation during pregnancy as they participate in the ICAN QUIT in pregnancy pilot step-wedge trial. Women Birth..

[15] Passey ME, Sanson-Fisher RW, Stirling JM (2014). Supporting pregnant Aboriginal and Torres Strait islander women to quit smoking: views of antenatal care providers and pregnant indigenous women. Matern Child Health J.

[16] Twyman L, Bonevski B, Paul C, Bryant J (2014). Perceived barriers to smoking cessation in selected vulnerable groups: a systematic review of the qualitative and quantitative literature. BMJ Open.

[17] Hunter New England Local Health District. HNELHD Internet Home Page. http://www.hnehealth.nsw.gov.au/Pages/home.aspx. Accessed 13th June, 2018.

[18] NSW Ministry of Health (2018). Tobacco and Smoking - Quit for New Life.

[19] Bursac Z, Gauss CH, Williams DK, Hosmer DW (2008). Purposeful selection of variables in logistic regression. Source Code Biol Med.

[20] Miller CL, Wakefield M, Roberts L (2003). Uptake and effectiveness of the Australian telephone Quitline service in the context of a mass media campaign. Tobacco Control.

[21] Gould GS, Munn J, Watters T, McEwen A, Clough AR (2013). Knowledge and views about maternal tobacco smoking and barriers for cessation in Aboriginal and Torres Strait islanders: a systematic review and meta-ethnography. Nicotine Tob Res.

[22] Tzelepis F, Daly J, Dowe S, Bourke A, Gillham K, Freund M (2017). Supporting Aboriginal women to quit smoking: antenatal and postnatal care providers’ confidence, attitudes, and practices. Nicotine Tob Res.

[23] Stead LF, Perera R, Bullen C, Mant D, Hartmann-Boyce J, Cahill K, Lancaster T. Nicotine replacement therapy for smoking cessation. Cochrane Database Syst Rev. 2012;(11). Art. No.: CD000146. 10.1002/14651858.CD000146.pub4. 10.1002/14651858.CD000146.pub4 23152200

[24] Coleman T, Chamberlain C, Davey MA, Cooper SE, Leonardi-Bee J. Pharmacological interventions for promoting smoking cessation during pregnancy. Cochrane Database Syst Rev. 2015;(12). Art. No.: CD010078. 10.1002/14651858.CD010078.pub2. 10.1002/14651858.CD010078.pub2 26690977

[25] Dempsey D, Jacob P, Benowitz NL (2002). Accelerated metabolism of nicotine and cotinine in pregnant smokers. J Pharmacol Exp Ther.

[26] Rebagliato M, Bol F (1998). du V. Florey C, et al. variations in cotinine levels in smokers during and after pregnancy. Am J Obstet Gynecol.

[27] van den Brand FA, Nagelhout GE, Reda AA, Winkens B, Evers SMAA, Kotz D, van Schayck OCP. Healthcare financing systems for increasing the use of tobacco dependence treatment. Cochrane Database Syst Rev. 2017;(9). Art. No.: CD004305. 10.1002/14651858.CD004305.pub5. 10.1002/14651858.CD004305.pub5 PMC6483741 28898403

[28] Hunter New England Local Health District. eMaternity-Archetype Report - Accepted AMIHS clients. Women identified as Aboriginal, Torres Strait Islander or Aboriginal/Torres Strait Islander. Accessed 15/12/2020.

[29] Thomas DP, Briggs VL, Couzos S (2015). Research methods of talking about the smokes: an international tobacco control policy evaluation project study with Aboriginal and Torres Strait islander Australians. Med J Aust.

