# Supplementary material for: Acceptance of smoking cessation support and quitting behaviours of women attending Aboriginal Maternal and Infant Health Services for antenatal care
Source: BMC Pregnancy Childbirth. 2021 Jan 26;21:85. doi: 10.1186/s12884-021-03569-z (PMC7836151; doi:10.1186/s12884-021-03569-z)
Supplement: Supplementary file 1 — Additional file 1. [file 12884_2021_3569_MOESM1_ESM.docx]

# HNELHD AMIHS Client Survey - Computer Assisted Telephone Interview Script

Intro 4-Select Interviewer: Do you identify as Aboriginal or Torres Strait Islander?

| Yes, Aboriginal | 1 | Go to Intro 5 |
| --- | --- | --- |
| Yes, Torres Strait Islander | 2 | Go to Intro 5 |
| Yes, both Aboriginal and Torres Strait Islander | 3 | Go to Intro 5 |
| No, neither | 4 | Q1 |
| Don’t know | 5 | Q1 |
| Refused | .R | Go to Intro 5 |

# CONT Intro 5: Would you prefer for one of our Aboriginal staff to call you back or are you happy to complete the survey now?

| Call back with Aboriginal staff member | 1 | SCHEDULE CALL BACK |
| --- | --- | --- |
| Continue with survey | 2 | Go to Q1 |
| Refused | .R | END |

INFO1 Please keep in mind that you don’t have to answer all the questions in the survey if you don’t want to, particularly if there’s any questions you don’t feel comfortable answering, just let me know and I can move on to the next question.

**All the questions in the survey relate to the visits you had with <local AMIHS> during your pregnancy. Lots of women we are phoning are being seen by other services and may be seeing their child and family health service for bub checks currently, but when you’re answering these questions, please think back to the times you were seen by <local AMIHS>.**

| **Questions** |  |  | |  |  |  | |  |  |
| --- | --- | --- | --- | --- | --- | --- | --- | --- | --- |
| 1 VISIT | **When <local**  **AMIHS> provided** | 1. Only 1 | | 2. 2-4 visits | 3. 5 or more visits | 888. Don’t know | | .R Refused |  |
|  | **care to you during** |  | |  |  |  | |  |  |
|  | **your pregnancy,** |  | |  |  |  | |  |  |
|  | **about how many** |  | |  |  |  | |  |  |
|  | **visits did you** |  | |  |  |  | |  |  |
|  | **have?** |  | |  |  |  | |  |  |
| 2  LAST  **TABLE** | **When did you last access the service?** | days | | weeks | months |  | |  |  |
| 3  ASKED | **At your visits, were you asked if you smoked?** | 1. Yes (Go to Q4) | | 2. No. They didn’t ask (Go to Q4) | 3. No. Because they already knew I smoked  (Go to Q4) | 888. Don’t know  (Go to Q4) | | .R Refused to respond  (Go to Q4) |  |
| 4  QLOFF | **Was Quitline offered to you?** | 1. Yes (Go to Q5) | | 2. No, it wasn’t offered (Go to Q12) | 888. Don’t know (Go to Q12) | .R Refused to respond  (Go to Q14) | |  |  |
|  |  |  | |  |  |  | |  |  |
| 5 | **Did you accept** | 1. Yes | | 2. No | 888. Don’t know | .R Refused to | |  |  |
| ACOFF | **the offer for** | (Go to Q7) | | (Go to Q6) | (Go to Q13) | respond | |  |  |
|  | **Quitline to call** |  | |  |  | (Go to Q13) | |  |  |
|  | **you?** |  | |  |  |  | |  |  |
| 6  NAOFF | **What were the reasons you didn’t want Quitline to call you?** | 1.I wasn’t interested in quitting smoking | | 2.I didn’t think it would help me | 3. I didn’t want to receive phone calls from the quitline | 4. I prefer face to face | | 5. Didn’t know what the Quitline was | 6. Other– open text box |
|  |  | 888. Don’t know | | .R Refused to respond |  |  | |  |  |
| 7  TKQL | **Did you end up talking with anyone from Quitline?** | 1. Yes (Go to Q8) | | 2. No  (Go to Q14) | 888. Don’t know (Go to Q14) | .R Refused to respond  (Go to Q14) | |  |  |
| 8  **NUM**  CALLS | **About how many calls did you receive?** | (number) (Go to Q9) | | 888. Don’t know (Go to Q9) | .R Refused |  | |  |  |
| 9 | **Were you offered** | 1. Yes | | 2. No | 888. Don’t know | .R Refused to | |  |  |
| OFFAC | **to talk with an** | (Go to Q10) | | (Go to Q11) | (Go to Q11) | respond | |  |  |
|  | **Aboriginal** |  | |  |  | (Go to Q11) | |  |  |
|  | **counsellor?** |  | |  |  |  | |  |  |
| 10  ACACO | **Did you accept that offer?** | 1. Yes  (Go to Q11) | | 2. No  (Go to Q11) | 888. Don’t know (Go to Q11) | .R Refused to respond  (Go to Q11) | |  |  |
| 11  INFO2 | **Next I’m going to read out 4 statements about your experience with the Quitline and I’d like you to tell me whether you: ‘strongly agree’ ‘agree’ ‘disagree’ ‘strongly disagree’ or are ‘neutral’.** | | | | | | | | |
| 11a. EXQLa | **Speaking with the Quitline encouraged me to try quitting smoking.**  **Do you ……** | 1. Strongly agree | | 2. Agree | 3. Neither agree nor disagree | 4. Disagree | | 5. Strongly disagree | .R Refused |
| 11b.  EXQLb | **Speaking with the Quitline helped me to understand my cravings to smoke, and to deal with them better.**  **Do you…..** | 1. Strongly agree | | 2. Agree | 3. Neither agree nor disagree | 4. Disagree | | 5. Strongly disagree | .R Refused |
| 11c.  EXQLc | **It was easy to talk about smoking and quitting with the Quitline.**  **Do you…..** | 1. Strongly agree | | 2. Agree | 3. Neither agree nor disagree | 4. Disagree | | 5. Strongly disagree | .R Refused |
| 11d.  EXQLd | **Speaking with the Quitline helped me to stay off the smokes.**  **Do you….** | 1. Strongly agree | | 2. Agree | 3. Neither agree nor disagree | 4. Disagree | | 5. Strongly disagree |  |
| 12  WAOQL | **If you had have been offered Quitline, would you have accepted the**  **offer?** | 1. Yes  (Go to Q14) | | 2. No  (Go to Q13) | 888. Don’t know (Go to Q14) | .R Refused (Go to Q14) | |  |  |
| 13 | **Why would’t you want Quitline to call you?** | 1.I wasn’t interested in quitting smoking | | 2.I didn’t think it would help me | 3. I didn’t want to receive phone calls from the quitline | 4. I prefer face to face | | 5. Didn’t know what the Quitline was | 6. Other– open text box |
|  |  |  | | 888. Don’t know | .R Refused |  | |  |  |
| INFO3  Preamble | **We’ll go on now with some questions about patches, gum, spray and other products that you might have used to help you quit.** | | | | | | | | |
| 4  PINRT | **Did the staff at**  **<AMIHS> explain to you how products such as patches and, gum could help you to quit?** | 1. Yes  (Go to Q15) | | 2. No  (Go to Q15) | 888. Don’t know (Go to Q15) | .R Refused (Go to Q15) | |  |  |
| 15 | **Did the staff at**  **<AMIHS> explain to you that using products such as patches and, gum would be less harmful for bub than continuing to**  **smoke?** | 1. Yes  (Go to Q16) | | 2. No  (Go to Q16) | 888. Don’t know (Go to Q16) | .R Refused (Go to Q16) | |  |  |
| 16  OFNRT | **Were you offered free gum, patches or other nicotine products to help you quit smoking?** | 1. Yes  (Go to Q17) | | 2. No  (Go to Q28) | 888. Don’t know (Go to Q28) | .R Refused (Go to Q30) | |  |  |
| 17  ACNRT | **Did you accept any of those products?** | 1. Yes  (Go to Q19) | | 2. No  (Go to Q18) | 888. Don’t know (Go to Q30) | .R Refused (Go to Q30) | |  |  |
| 18  NANRT | **What were your reasons for not accepting any of the products (such as gum, patches)** | 1.I wasn’t interested in quitting smoking (Go to Q30) | | 2.I didn’t think it would help me (Go to Q30) | 3. Tried NRT in the past and it didn’t work/I didn’t like it (Go to Q30) | 4. I was worried it might not be safe to use whilst pregnant (harmful for baby)  (Go to Q30) | | 5.I wouldn’t feel comfortable using it/embarrassing (Go to Q30) | 6. Other – open text box (Go to Q30) |
|  |  | 888. Don’t know (Go to Q30) | | .R Refused (Go to Q30) |  |  | |  |  |
| 19 | **Were you given instructions on**  **when and how to use the products?** | 1. Yes  (Go to Q20) | | 2. No  (Go to Q20) | 888. Don’t know (Go to Q20) | .R Refused (Go to Q20) | |  |  |
| 20  WTNRT | **What type/s did**  **you use?** | 1. Spray/Mist | | 2. Lozenge | 3. Gum | 4. Inhalator  /Inhaler | | 5. Patches | 6. Other –  open text box |
|  |  | 7. Don’t know | | .R Refused |  |  | |  |  |
| 21a  UNRT  **New Loop:** | **Are you still using**  **^ ////^ now? Question 21 – 26 (repeat for each type of NRT** | 1. Yes  **(Go to 22)** | | 2. No | 888. Don’t know |  | |  |  |
| 22  USENRT | **How long did you use/have you been using ^insert Q20 response^?** | 1. Less than one day | | 2. 1-3 days | 3. 4-7 days | 4. 8-14 days | | 5. More than 2 weeks – 4 weeks | 6. More than 4 weeks - 8 weeks |
|  |  | 7. More than 8 weeks - 12 weeks | | 8. more than 12 weeks | 888. Don’t know | .R Refused | |  |  |
| 23 | **When you** | 1. I had quit | | 2. I didn’t like the taste | 3. I didn’t like the | 4. I ran out | | 5.It didn’t help | 6. I didn’t feel |
| STNR | **stopped using** | successfully/ | |  | side effects eg) |  | | with the | comfortable |
|  | **^insert Q20** | Didn’t think I | |  |  |  | | cravings/wasn’t | using |
|  | **response^ what** | needed it | |  |  |  | | working | it/embarrassed |
|  | **was the reason/s** | anymore | |  |  |  | |  |  |
|  | **you stopped?** |  | |  |  |  | |  |  |
|  |  | 7 Started smoking again | | 8. Forgot to take it | 9. Someone else took it/ Shared it with my family or friends | 10. Other – opens text box | | 888. Don’t know | .R Refused |
| INFO4  Preamble | **For the next three statements, can you tell me again if you ‘strongly agree’ ‘agree’ ‘disagree’, ‘strongly disagree’ or are ‘neutral’ (5**  **point scale)** | | | | | | | | |
| 24a.  ENCOUR | **Using ^insert Q20 response^ encouraged me to try quitting.**  **Do you….** | 1. Strongly Agree | | 2. Agree | 3. Neither agree nor disagree | 4. Disagree | | 5. Strongly disagree | .R Refused |
| 24b.  COPE | **Using ^insert Q20 response^**  **helped me cope with the cravings to smoke, and to**  **deal with them better. Do you….** | 1. Strongly Agree | | 2. Agree | 3. Neither agree nor disagree | 4. Disagree | | 5. Strongly disagree | .R Refused |
| 24c.  STAYOF | **Using ^insert Q20 response^**  **helped me to stay off the smokes.**  **Do you…** | 1. Strongly Agree | | 2. Agree | 3. Neither agree nor disagree | 4. Disagree | | 5. Strongly disagree | .R Refused |
|  | **You indicated that you used more than one type….** | | | | | | | | |
| 25 | **Did you use these products in**  **combination (at the same time) or**  **at different times?** | 1. In combination (Go to Q26) | | 2. At different timeframes (Go to Q30) | 3. I don’t know (Go to Q30) | .R Refused (Go to Q30) | |  |  |
| 26 | **What forms did you use together?** | | 1. Patches and gum | 2. Patches and spray | 3. Patches and inhalator | 4. Patches and lozenges | | 5. Spray and gum | 6. Spray and inhalator |
|  |  | | 7. Spray and lozenges | 8. Gum and inhalator | 9. Gum and lozenges | 10. Inhalator and lozenges | | 11. Patches  and more than 1 oral form | 12. More than  2 oral forms only |
|  |  | | 13. Other – opens text box | 888. Don’t know | .R Refused |  | |  |  |
| 27 | **Why did you use a combination?** | | 1. It was recommended by friend or relative | 2. It was recommended to me by a health professional/ midwife/  Aboriginal Health Worker | 3. I found one wasn’t enough to reduce cravings/ not strong enough/still wanted  to smoke when I used one | 4. Don’t know | | 5. Other – opens text box | .R Refused |
| 28  WAONRT | **If you had have been offered free patches, gum, lozenges, oral mist/ spray or inhalers would you have accepted them?** | | 1. Yes  (Go to Q30) | 2. No  (Go to Q29) | 888. Don’t know (Go to Q30) | .R Refused (Go to Q30) | |  |  |
| 29 | **What would be the reason for not accepting?** | | 1.I wasn’t interested in quitting smoking (Go to Q30) | 2.I didn’t think it would help me  (Go to Q30) | 3. Tried NRT in the past and it didn’t work/I didn’t like it (Go to Q30) | 4. I was worried it might not be safe to use whilst pregnant  Go to Q30) | | 5.I wouldn’t feel comfortable using it/embarrassing (Go to Q30) | 6. Other – open text box (Go to Q30) |
|  |  | | 888. Don’t know | .R Refused |  |  | |  |  |
| INFO5  Preamble | **The next questions ask about follow up support you received from <service name> to make changes to smoking. This could have been in the form of specific appointments to talk about quitting, phone calls or texts or time set aside in your usual appointments.** | | | | | | | | |
| 30 OFFUS | **Again, this is only about when you were seeing**  **<local AMIHS> team. Were you**  **offered follow up support?** | | 1. Yes  (Go to Q31) | 2. No, I wasn’t offered a follow-up(Go to Q35) | 888. Don’t know (Go to Q35) | .R Refused (Go to Q37) | |  |  |
| 31 ACFUS | **Did you accept follow up support**  **around quitting?** | 1. Yes  (Go to Q33) | | 2. No  (Go to Q32) | 888. Don’t know (Go to Q37) | .R Refused (Go to Q37) | |  |  |
| 32 NAFUS | **What were your reasons for not accepting follow- up support to talk about quitting?** | 1 I wasn’t interested in quitting smoking (Go to Q37) | | 2. I didn’t think it would help me  (Go to Q37) | 3. I didn’t feel comfortable talking with the AMIHS staff about making changes to my smoking  (Go to Q37) | 4. Other – open text box  (Go to Q37) | | 888. Don’t know  (Go to Q37) | .R Refused to respond  (Go to Q37) |
| 33 SOFUS | **What sort of follow up support were you given?** | 1. Text messaging | | 2. Phone calls | 3. Face to face visits to talk only about quitting/making changes to my smoking | 4. Face to face follow -up about quitting in my usual appointments | | 5. None | 888. Don’t know |
|  |  | .R Refused | |  |  |  | |  |  |
| 34 INFO6 | **These statements are about your experience with follow-up support and again I’d like you to tell me whether you ‘agree’**  **‘disagree’ or are ‘not sure’.** | | | | | | | | |
| 34a.  FUSUPa | **Follow up support from the <AMIHS team> encouraged me to try quitting.**  **Do you….** | 1. Strongly Agree | | 2. Agree | 3. Neither agree nor disagree | 4. Disagree | | 5. Strongly disagree | .R Refused |
| 34b.  FUSUPb | **Follow up support helped me to understand my cravings to smoke, and to deal with them**  **better**  **Do you….** | 1. Strongly Agree | | 2. Agree | 3. Neither agree nor disagree | 4. Disagree | | 5. Strongly disagree | .R Refused |
| 34c.  FUSUPc | **I felt comfortable talking about smoking and quitting with the**  **<AMIHS team>.**  **Do you….** | 1. Strongly Agree | | 2. Agree | 3. Neither agree nor disagree | 4. Disagree | | 5. Strongly disagree | .R Refused |
| 34d.  FUSUPd | **Follow up support from the staff helped me to stay**  **off the smokes.** | 1. Strongly Agree | | 2. Agree | 3. Neither agree nor disagree | 4. Disagree | | 5. Strongly disagree | .R Refused |
| 35  WAOFUS | **If you had have been offered follow-up, support to talk about quitting, such as specific appointments to talk about quitting, phone calls or texts, time set aside in your usual appointments,**  **would you have accepted it?** | 1. Yes  (Go to Q37) | | 2. No  (Go to Q36) | 888. Don’t know (Go to Q37) | R. Refused (Go to Q37) | |  |  |
| 36 | **Why wouldn’t you have wanted follow up support from <AMIHS> to help make**  **changes to your smoking?** | 1. I wasn’t interested in quitting smoking (Go to Q37) | | 2. I didn’t think it would help me  (Go to Q37) | 3. I didn’t feel comfortable talking with the AMIHS staff about making changes to my smoking (Go to Q37) | 4. Other – open text box  (Go to Q37) | | 888. Don’t know  (Go to Q37) | .R Refused to respond  (Go to Q37) |
|  |  | .R Refused | |  |  |  | |  |  |
| 37 | **During your visits, did the staff offer to measure your**  **carbon monoxide levels using a smokerlyser machine?** | 1. Yes  (Go to Q38) | | 2. No  (Go to Q39) | 888. Don’t know (Go to Q39) | R. Refused (Go to Q39) | |  |  |
| 38 | **Did it encourage you to make changes to your**  **smoking?** | 1. Yes  (Go to Q39) | | 2. No  (Go to Q39) | 888. Don’t know (Go to Q39) | R. Refused (Go to Q39) | |  |  |
| INFO7  Preamble | **The next few questions ask about quitting. When we talk about quitting for this survey we mean deliberately deciding to stop smoking for 24hours or more in an attempt to give up smoking.** | | | | | | | | |
| 39 QUIT1 | **During the time that you were seeing <local AMIHS> did you**  **ever quit for 1 day or more?** | 1. Yes  (Go to Q40) | | 2. No  (Go to Q42) | 888. Don’t know (Go to Q42) | R. Refused (Go to Q42) | |  |  |
| 40 TIMEQ  NUM | **How many times did you quit smoking for 1 day**  **or more during that time?** | Number: (Go to Q41) | | 888. Don’t know (Go to Q42) | .R Refused (Go to Q42) |  | |  |  |
| 41  ATTPQ NUM | **Of the quit attempts that you made, what was the longest time**  **you went without a cigarette?** | Days: (Go to Q42) | | Months: (Go to Q42) | Still quit (time frame)  (Go to Q42) | 888. Don’t know  (Go to Q42) | | .R Refused (Go to Q42) |  |
| 42  CHANG  MULT | **During the time**  **you were seeing <local AMIHS> did you make any other changes to your smoking?** | 1.Cut down the number of cigarettes I smoked (Go to Q43) | | 2. Stopped smoking inside the house (Go to Q43) | 3. Stopped smoking in the car (Go to Q43) | 4. Stopped smoking at work  (Go to 43) | | 5. Started buying lighter/different brand cigarettes  (Go to Q43) | 6. Stopped smoking in public  (Go to Q43) |
|  |  | 7. Only smoke at night  (Go to Q43) | | 8. Other. Opens text box  (Go to Q43) | 888. Don’t know (Go to Q43) | .R Refused (Go to Q43) | |  |  |
| 43 | **During the time you were seeing AMIHS did you use any other methods than the ones we’ve talked about to help you to quit smoking?** | 1. Yes (Go to 44) | | 2. No  (Go to Q45) | 888. Don’t know (Go to Q45) | .R Refused (Go to Q45) | |  |  |
| 44 | **What other methods did you use?** | 1. E-cigarettes (Go to 45) | | 2. Hypnosis (Go to 45) | 3. Meditation (Go to 45) | 4. Group/ workshop (Go to 45) | | 5. DVDs (Go to 45) | 6. Other. Opens text box  (Go to 45) |
|  |  | 888. Don’t know  (Go to 45) | | .R Refused (Go to 45) |  |  | |  |  |
| 45  Preamble | **The following questions are about <local AMIHS> staff providing support to clients to quit smoking. I’d like you to tell me whether**  **you ‘agree’ ‘disagree’ or are ‘not sure’ about the following statements** | | | | | | | | |
| 45 | **I expect to be asked about smoking during my pregnancy care** | 1. Agree | | 2. Disagree | 3. Not sure | .R Refused | |  |  |
| 46 | **It is important for**  **<local AMIHS> staff to help smoking clients to quit.** | 1. Agree | | 2. Disagree | 3. Not sure | .R Refused | |  |  |
| SMOKE | **The next questions are about your smoking now.** | | | | | | | | |
| 47  SMOKE | **Do you currently smoke any tobacco products?** | 1.Daily  (Go to Q48) | | 2.At least once a week (Go to Q48) | 3. Less often than once a week  (Go to Q48) | 4. Not at all (Go to Q50) | | .R Refused |  |
|  |  |  | |  |  |  | |  |  |
| 48 | **How soon after** | minutes | | hours |  |  | |  |  |
| FSMK | **you wake up do** |  | |  |  |  |  |  |  |
|  | **you smoke your** |  | |  |  |  |  |  |  |
|  | **first cigarette?** |  | |  |  |  |  |  |  |
| 49 MANY | **How many cigarettes per day do you smoke?** | Number: | |  |  |  | |  |  |
| 50 PARTC | **Have you smoked at least part of a cigarette or used any other forms of tobacco, for example, pipes or cigars in the last 7 days?** | 1. Yes (Go to 52) | | 2. No  (Go to 51) | 888. Don’t know  (Go to 51) | .R Refused (Go to 51) | |  |  |
| 51 SINC | **How long is it since you last smoked a cigarette or used any other forms of**  **tobacco?** | days | | weeks | Months | years | |  |  |
| 52 true | **Thinking about quitting, which is true for you at the moment?** | 1. I Never expect to quit | | 2. I will quit in the next 30 days | 3. I will quit in the next 6 months | 4. I might quit in the future, but not in the next 6 months | | 888. Don’t know | .R Refused |
| 53 | **What are the main things stopping you from becoming a non-smoker?** | 1. Like smoking/being a smoker | | 2. Quitting is too hard | 3. Stress/need it to cope | 4. Everyone/ people around me smoke | | 5. No family support | 6. No help to quit / access to help to quit |
|  |  | 7. Other. Opens text box | | 888. Don’t know | .R Refused |  | |  |  |
| 54  HOUSE | **How many other people in your household smoke, if any?** | Number: | |  |  |  | |  |  |
| 55  FAMSP | **During your <local AMIHS> visits, was quit support offered to your family or household members who smoked i.e. free NRT vouchers?** | 1. Yes (Go to 56) | | 2. No  (Go to Q57) | 888. Can’t recall / Don’t know  (Go to Q57) | .R Refused (Go to Q57) | |  |  |
| 56  SOFF | **What kind of support was offered?** | 1. NRT Voucher | | 2. Quitline referral | 3. Other – open text box | 888. Don’t know | | .R Refused |  |
| 57  SITH | **Which best describes the situation at home in relation to smoking?** | 1. There is no smoking inside the house at all | | 2. Smoking is limited to part of the house where the children rarely go | 3. Smoking does not occur in children’s bedrooms but occurs in other common rooms of the house eg lounge, kitchen | | 4. Smoking is allowed in any room | 888. Don’t know | .R Refused |
| 58 | **When would you make an exception to this? Would it be..** | 1. Never | | 2. When children are not at home | 3. When children are in bed | | 4. During really hot really cold weather | When smoking friends or family visit. | 888. Don’t know |
| 59  Preamble | **To help us plan new ways to support mums to quit smoking we are interested in whether you would use the following strategies?** | | | | | | | | |
|  |  | **Very likely** | | **Likely** | **Unsure** | | **Unlikely** | **Very unlikely** |  |
| 59 | **Quit app on your**  **smartphone** | **1** | | **2** | **3** | | **4** | **5** |  |
| 60 | **Text messaging**  **quit support** | **1** | | **2** | **3** | | **4** | **5** |  |
| 61 | **Facebook page to support mums quitting** | **1** | | **2** | **3** | | **4** | **5** |  |
| 62 | **A quit smoking counsellor appointment on video link up through your smartphone or ipad.** | **1** | | **2** | **3** | | **4** | **5** |  |
| 63 | **Face to face**  **support groups** | **1** | | **2** | **3** | | **4** | **5** |  |
| Preamble | **These last couple of questions help us to identify any patterns, and ask a little about your background, but again, it will not identify you.** | | | | | | | | |
| 64 AGE | **Could you please tell me how old**  **you are today?** | years | |  |  | |  |  |  |
| 65 EDUC | **Which of these is true for you?** | 1. I completed primary school | | 2. I completed years 7 to 9 | 3. Finished high school with a School Certificate (Intermediate, Year 10, 4th Form) | | 4. I finished school with an Higher School Certificate (Year 12, 6th Form) | 5. I have a TAFE certificate or diploma | 6. I have a University degree, College of Advanced Education, etc. |
|  |  | 888. Don't know | | .R Refused |  | |  |  |  |
| 66  RELAT | **Which of these is true for you?** | 1. Single – living without a partner | | 2. Single - Living with other family members/support | 3. Live with my partner or married | | 4. Other | .R Refused |  |
| 67  RESU | **That almost completes the survey, thanks for answering our questions. Just before you go, Would you like to receive a summary of the results of this**  **survey when they are finalised?** | 1. Yes  (Go to 47 if smoke in (1,2,3)  or  (Go to 48 if smoke in (4 ,.R) | | 2. No  (Go to 47 if smoke in (1,2,3  or  (Go to 49 if smoke in (4 ,.R) | 3.Don’t know  (Go to 47 if smoke in (1,2,3  or  (Go to 49 if smoke in (4 ,.R) | | .R Refused (Go to 47 if smoke in (1,2,3  or  (Go to 49 if smoke in (4  ,.R) |  |  |
| 68  PACK | **We have free quit smoking packs available to all participants, would you like us to send you one** | 1. Yes (Go to 48) | | 2. No  (Go to 48 if RESU=1) or  if RESU in (2,3,.R)  and PACK in (2,3,.R) go to 49 | 3.Don’t know  (Go to 48 if RESU=1)  if RESU in (2,3,.R)  and PACK in (2,3,.R) go to 49 | .R Refused (Go to 48 if RESU=1)  if RESU in (2,3,.R) and  PACK in (2,3,.R) go to 49 | |  |  |
